# Agenesis of internal carotid artery associated with isolated growth hormone deficiency: a case report and literature review

**DOI:** 10.1186/s12902-015-0037-y

**Published:** 2015-10-19

**Authors:** Stefano Stagi, Giovanna Traficante, Elisabetta Lapi, Marilena Pantaleo, Sabrina Becciani, Marzia Mortilla, Salvatore Seminara, Maurizio de Martino

**Affiliations:** Paediatric Endocrinology Unit, Health Sciences Department, University of Florence, Anna Meyer Children’s University Hospital, Viale Pieraccini 24, 50139 Florence, Italy; Genetics and Molecular Medicine Unit, Anna Meyer Children’s University Hospital, Florence, Italy; Radiology Unit, Anna Meyer Children’s University Hospital, Florence, Italy

## Abstract

**Background:**

Agenesis of the internal carotid artery (ICA) is a rare congenital abnormality, sporadically reported to be associated with a combined congenital hypopituitarism. Nevertheless, only a few cases have been extensively described, and none of these have been characterized by an isolated growth hormone (GH) deficiency.

**Case presentation:**

Here, we describe a 17-year old boy referred to our hospital for fatigue, decreased muscle strength and severe headache reported after the cessation of rhGH treatment for a GH deficiency diagnosed at the age of 2 years and 3 months. Magnetic resonance imaging (MRI) showed an adenohypophyseal hypoplasia with a lack of posterior pituitary hyperintensity, whereas MRI angiography indicated the absence of a normal flow void in the left ICA. Endocrinological tests confirmed the GH deficiency (GH peak after growth-hormone-releasing hormone (GHRH) + arginine: 2.42 ng/mL) with a very low IGF-I value (31 ng/mL) and normal function of other pituitary axes.

**Conclusion:**

To the best of our knowledge this is the first confirmed case of an isolated GH deficiency in a patient with ICA agenesis. The presence of an isolated pituitary deficit is unlike to be considered only as an effect of hemodynamic mechanism, suggesting a role for genetic factor(s) as a common cause of these two rare birth defects. Further studies could clarify this issue and the underlying mechanisms to better understand the etiopathogenetic characteristics of this disorder.

**Electronic supplementary material:**

The online version of this article (doi:10.1186/s12902-015-0037-y) contains supplementary material, which is available to authorized users.

## Background

Agenesis, aplasia and hypoplasia of the internal carotid artery (ICA) are rare congenital vascular malformations [1]. Of these, agenesis of the ICA is an extremely rare vascular anomaly occurring in less than 0.01 % of the population [2]. A 3:1 left side predominance in the ICA agenesis has been reported [1].

Agenesis of the ICA is rarely associated with congenital hypopituitarism (CH) [3]. CH is also a very rare congenital manifestation presenting either as an isolated hormone deficiency—the most common of which is isolated growth hormone deficiency (IGHD)—or multiple pituitary hormone involvement (combined pituitary hormone deficiencies (CPHD)) [4].

The etiology of this association is not clear: even if the blocking of a unilateral blood supply might cause the pituitary hypoplasia, a possible genetic cause, such as complex neural crest differentiation and/or migration disorders, cannot be excluded.

To the best of our knowledge, no more than 12 cases of CPHD associated with ICA have been reported [2, 5–14] (Table 1). However, a case of transient IGHD has been also described even though it may be caused by a reduced flow because of a focal stenosis in the right distal internal carotid artery and right middle cerebral artery due to a moyamoya [15]. Given the rarity of these two conditions, the association is unlikely to be causal, although this possibility cannot be excluded. In this report, we describe for the first time a 17-year-old boy with a confirmed diagnosis of IGHD and we review the literature and discuss the etiology of this association.

## Case report

The patient was a 17-year-old boy on rhGH therapy for 14 years because of a GH deficiency that was diagnosed at the age of 2 years and 3 months. Therapy was stopped 9 months before the beginning of symptoms—fatigue, decreased muscle strength and severe headache. However, the patient had not come for retesting one month after discontinuation of rhGH treatment.

The propositus was the first child of non-consanguineous Italian parents. The mother was in treatment for post-partum depression. The maternal grandfather was in treatment for autoimmune hyperthyroidism.

The mother’s height was 152 cm and she had menarche at the age of 13; the father’s height was 168 cm and he had a normal development. The target height was 166.0 ± 6.0 cm (−1.70 SDS). All auxological data were normalized for chronological age by conversion to standard deviation scores (SDS) that were calculated according to the following formula: patient value - mean of age-related reference value/standard deviation of the age-related reference value [16].

The child was born at 40 weeks of gestation with an uncomplicated delivery. The birth weight was 2970 g (−1.15 SDS), length at birth was 50 cm (−0.30 SDS), and his occipito-frontal head circumference (OFC) was 34 cm (−0.61 SDS). He had neonatal jaundice requiring phototherapy. The child’s neuromotor development was normal: he sat at 6 months, walked independently at 12 months, and began to use language at 14 months.

**Fig. 1:**
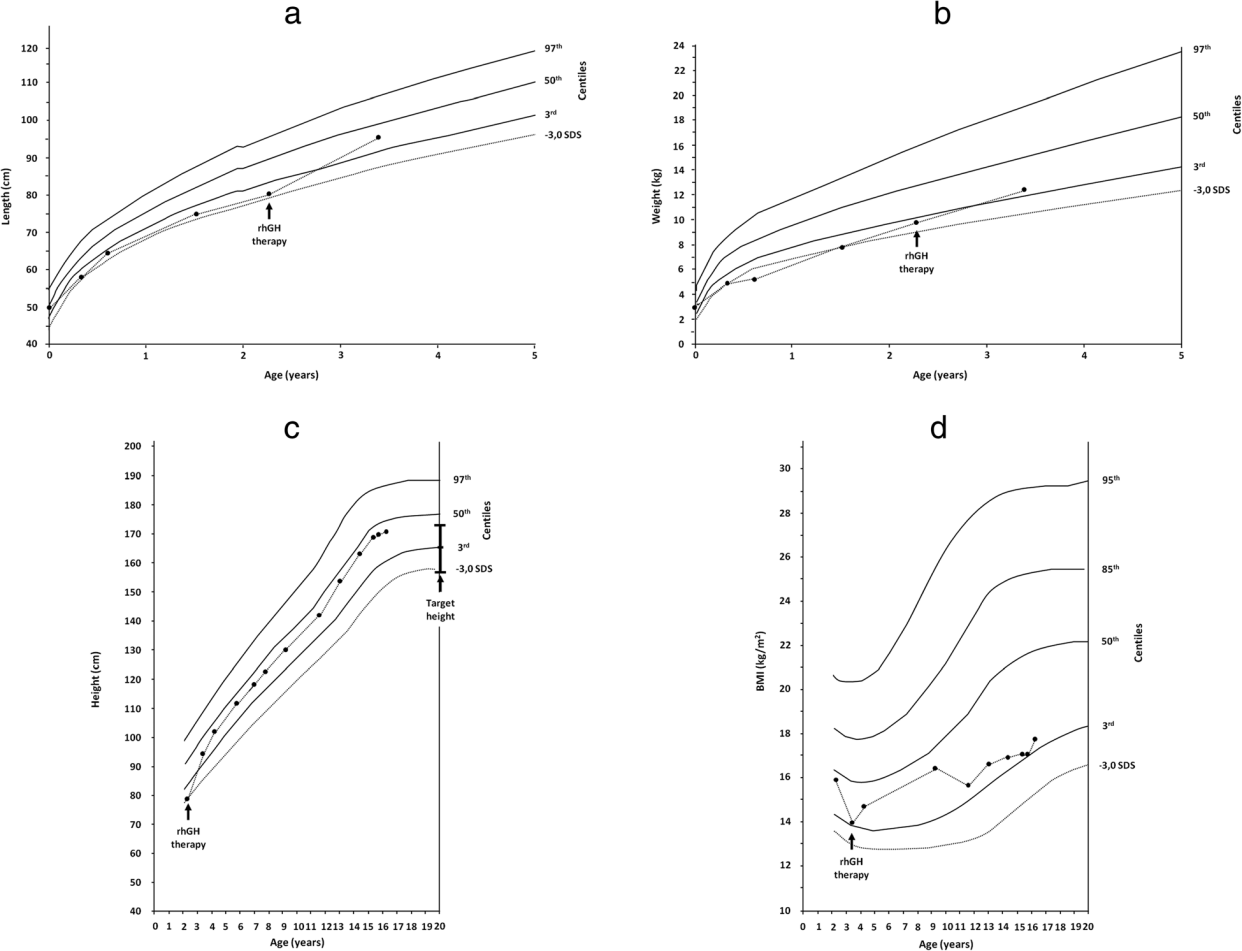
Length (**a**), weight (**b**), height (**c**) and body mass index (BMI; **d**) growth charts of the patient. The growth charts of the length and weight refer to the first 3 years 4 months of life. The arrows indicate the age at which growth hormone treatment was initiated (black arrow). The length and the weight were evaluated according the growth charts compiled by de Onis et al. [37]. The height and BMI were evaluated according the growth charts compiled by Cacciari et al. [17]

At 6 months, the patient exhibited very deficient growth (Fig. 1a and b). At 2 years and 3 months, he was admitted to our Paediatric Endocrine Unit for an endocrinological evaluation. His height, evaluated according the growth charts compiled by Cacciari *et al.* [17], was 78.6 cm (−2.79 SDS). His weight was 9.830 kg (−2.78 SDS), and his BMI was 15.91 (−0.28 SDS) (Fig. 1a-d). Extensive biochemical, endocrinological and metabolic examinations did not reveal any abnormalities with the exception of plasma concentrations of IGF-I and IGFBP-3 that were low for his age and sex: 27 ng/mL and 1.38 μg/mL, respectively. Thus, GH stimulation tests were performed and showed a reduced response after the clonidine (GH peak of 5.2 ng/mL) and insulin (glucose nadir 2.0 mmol/L at 30 min; GH peak of 3.9 ng/mL) tests. This revealed a GH deficiency (GHD). Bone age was assessed according to the Greulich and Pyle method [18], and it was delayed (1 year and 2 months versus a chronological age of 2 years and 3 months). Brain MRI of the hypophyseal region showed the presence of a pituitary hypoplasia (height 1.8 mm and width 2.7 mm). Consequently, hGH therapy (0.22 mg/kg/wk) was started.

The child responded very well to rhGH treatment (Fig. 1c) and at 3 years and 4 months old, his height was 94.5 cm (−1.14 SDS), weight 12.450 kg (−2.10 SDS), height velocity 15.06 cm/yr (5.05 SDS), and BMI 13.94 (−1.75 SDS). At 4 years and 3 months old, his height was 101.9 cm (−0.82 SDS), his weight was 15.300 kg (−1.24 SDS), his height velocity was 8.05 cm/yr (1.12 SDS), and his BMI was 14.73 (−0.82 SDS).

The onset of puberty occurred at 12 years old with normal onset, timing, tempo, and magnitude of pubertal changes [19]. He stopped rhGH treatment at 16 years and 3 months old when he reached the definitive stature [16]: his height was 170.8 cm (−0.64 SDS) and his weight was 51.800 kg (−1.56 SDS) with a BMI of 17.76 (−1.63 SDS) (Fig. 1c and d).

At 17 years old, his GH secretion was re-tested by the growth-hormone-releasing hormone (GHRH) + arginine test, which is a reliable test of ITT in retesting patients who had undergone GH treatment in childhood [20]. The test confirmed a severe GH deficiency (basal GH value of 0.18 ng/mL; GH peak of 2.42 ng/mL).

**Fig. 2:**
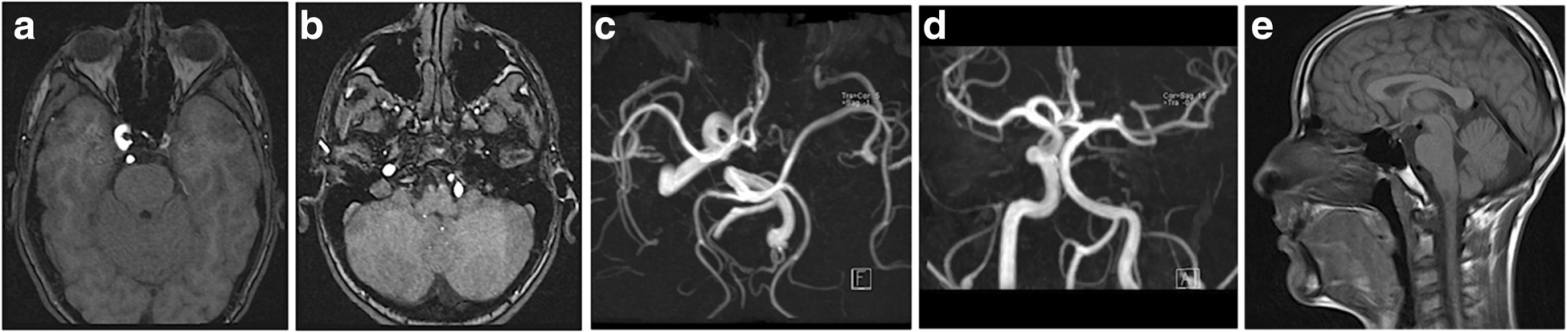
A MRI angiography time-of-flight (TOF) three-dimensional (3D) technique through the circle of Willis revealed agenesis of the intracranial portion of the left internal carotid artery (ICA). **a**. 3D TOF in the axial plane at the carotid siphon level shows agenesis of the left ICA. **b**. 3D TOF in the axial plane for the vertebrobasilar system shows the left vertebral artery and the compensatory hypertrophy. **c**. MRI angiography maximum intensity projection (MIP) reconstruction in the axial plane reveals agenesis of the intracranial portion of the left ICA. **d**. MRI angiography MIP reconstruction in the coronal plane highlights hypertrophy in the left vertebral collateral blood flow. **e**. Sagittal T1W TSE sequence shows adenohypophyseal hypoplasia (height 2.5 mm and width 3.5 mm) with a lack of posterior pituitary hyperintensity

MRI angiography was performed because of the severe growth hormone deficiency (GHD) and headache; it confirmed the presence of adenohypophyseal hypoplasia (height 2.5 mm and width 3.5 mm) with a lack of posterior pituitary hyperintensity. The MRI angiography also showed the absence of a normal flow void in the left ICA (Fig. 2).

No abnormal findings were noted in the electrocardiogram. Chromosomal studies of the peripheral lymphocyte cultures revealed a 46,XY normal male karyotype. An array of CGH analyses on the patient’s DNA was performed with an Agilent 60 K array platform with a resolution of approximately 100 kb. No genomic imbalances were detected. A complete examination was performed with particular attention to neurology, metabolism, and endocrinology (Table 2). The only abnormal result was a very low IGF-I value (31 ng/mL). In light of these results, the patient restarted rhGH therapy, and his symptoms at the time of referral disappeared.

**Table 1:** Review of endocrinological and anatomical characteristics of patients with congenital hypopituitarism and internal carotid malformations

	Sex	Age	Pituitary MRI	Internal carotid RMI	Other characteristics	Molecular studies	References
1	N	N	Not specified (except for normal posterior pituitary).	Absence of ICA and carotid canal.	Not specified.	NP	[14]
2	M	18 m	Pituitary hypoplasia.	Anomaly of right ICA.	TSH, GH, ACTH deficiency. Central diabetes insipidus. Possible hypogonadism.	NP	[13]
3	F	5 m	Absence of anterior pituitary and ectopic pituitary posterior lobe.	Absence of right common carotid artery, right ICA, right anterior cerebral artery.	TSH, ACTH and GH deficiency. No evidence of diabetes insipidus. Genitalia were normal. Single central maxillary incisor.	NP	[7]
4	M	23 year	Absence of anterior pituitary and ectopic pituitary posterior lobe.	Absence of right ICA and carotid canal and A1 segment of the right anterior cerebral artery.	Congenital microphthalmia with cataract and coloboma of the right eye, encephalocele. Hormonal substitution treatments not specified.	NP	[5]
5	M	37 year	Absence of anterior pituitary.	Hypoplasia of right ICA and carotid canal.	Amblyopia of the left eye caused by an optic nerve coloboma, encephalocele. Hormonal substitution treatments not specified.	NP	[5]
6	F	29 year	Pituitary hypoplasia and ectopic pituitary posterior lobe.	Absence of right ICA, carotid canal, and A1 segment of the right anterior cerebral artery.	TSH, ACTH, and GH deficiency. No evidence of diabetes insipidus. Hypogonadism. Chiari I malformation with syringomyelia.	NP	[9]
7	M	5 year	Absence of anterior pituitary and ectopic pituitary posterior lobe.	Absence of left ICA and carotid canal, A1 segment of the left anterior cerebral artery and the anterior communicating artery.	Retrognathia, microphallus, and cryptorchidism. TSH, ACTH, GH deficiency, but no evidence of diabetes insipidus. Possibile hypogonadism.	NP	[10]
8	M	11 year	Pituitary hypoplasia	Absence of left ICA and carotid canal; hypoplasia of A1 segment of left anterior cerebral artery	TSH, ACTH, GH deficiency. Central diabetes insipidus. Microphallus with possible hypogonadism.	NP	[6]
9	F	10 year	Hypoplastic anterior pituitary, flat sella turcica, absent pituitary stalk	Agenesis of the left ICA and the left carotid canal	GH, TSH, gonadotropin deficiency. No evidence of diabetes insipidus. Born out of a consanguineous marriage.	No *HESX1*, *LHX4*, *OTX2* mutations	[8]
10	F	7 month	Adenohypophyseal hypoplasia with a lack of posterior pituitary hyperintensity	Absence of the left ICA	Desaturation episodes, recurrent respiratory infections. Short hands and feet. GH, ACTH and TSH deficiency.	17q24.2 deletion	[3]
11	F	2 year	Adenohypophyseal hypoplasia with a lack of posterior pituitary hyperintensity	Absence of the right ICA	GH, TSH, and gonadotropin deficiency. No clinical evidence of diabetes insipidus.	NP	[11]
12	M	3 week	Absence of anterior pituitary with ectopic posterior pituitary	Absence of the left ICA and carotid canal	GH, TSH, ACTH and gonadotropin deficiency. Microphallus.	NP	[12]
13	M	17 year	Adenohypophyseal hypoplasia with a lack of posterior pituitary hyperintensity	Agenesis of the left ICA	GH deficiency. No other pituitary deficiencies. No clinical evidence of diabetes insipidus.	Normal array-CGH	Our case

*NP* not performed

**Table 2:** Laboratory results at left ICA agenesis diagnosis

	Value	Reference values		Value	Reference values
FreeT4, pmol/L	12.4	10.3–19.4	HbA1c, %	4.4	<5.5
TSH, mIU/L	1.20	0.40–4.0	Na^+^, mEq/L	138	135–145
Cortisol, μg/dL	16.8	5.0–19.0	K^+^, mEq/L	4.2	3.5–5.0
ACTH, ng/L	25.7	9.0–52.0	Cl^−^, mEq/L	101	95–105
Prolactin, mIU/L	271	87.0–392.0	Plasma osmolality, mOsm/kg	295	280–300
Total testosterone, ng/mL	378	270.0–1070.0	Urine Osmolality, mOsm/kg	1037	400–1100

*TSH* thyroid-stimulating hormone, *ACTH* adrenocorticotrophic hormone

## Discussion

ICA agenesis is a rare congenital abnormality that is very rarely associated with CH. All reported cases refer to patients with CPHD and ICA agenesis although many of these reports did not describe—or only poorly described—the possible associated endocrinological abnormalities and treatments. This had led many to hypothesize a vascular cause of pituitary disorders. Two different theories involving the hemodynamic mechanisms or disorders of differentiation and/or migration of the neural crest have been reported [2].

The ICA originates from the third aortic arch during embryogenesis. However, it is controversial whether the common and external carotids really originate from the same third aortic arch or from the aortic sac [21]. In patients presenting a vascular anomaly of the ICA, the blood supply of the affected side is partially or completely compensated by the vertebrobasilar or contralateral ICA system. This is characterized by the enlargement of a normally existing segment or presence of abnormal or persisting fetal arteries [22]. In most patients, this vascular anomaly is asymptomatic [23], and the rarity of a pediatric ICA agenesis diagnosis suggests that the arterial collateral pathways are initially sufficient to support cerebral perfusion [24]. However, some patients may experience recurrent headaches, blurred vision, hemiparesis, and hypopituitarism [25, 26] likely due to cerebrovascular insufficiency, compression by collateral vessels, or associated cerebral aneurysms [21]. Moreover, the symptoms reported by our patient were probably the result of both GHD (fatigue, decreased muscle strength, etc.) and ICA agenesis (headache).

Only one of the 13 patients previously described in the literature had MRI evidence of associated vascular defects. This patient had transient GH deficiency and was described by Quah *et al.* [15]. This patient’s pituitary defects and neurological symptoms may have been caused by reduced flow due to a focal stenosis of the other distal internal carotid artery and right middle cerebral artery due to a moyamoya [15]. However, the presence of a defect in the vascular perfusion is difficult to reconcile with the pituitary anatomical abnormalities and with the variability of the endocrinological defects reported including isolated GH deficiency or central diabetes insipidus (described only rarely) [6, 15].

The ICA is formed in embryos during the fourth week from the third aortic arch arteries and during the fifth and sixth weeks from the terminal segments of the dorsal aorta [27]. Conversely, the pituitary gland fully develops due to merging of the Rathke pouch and the infundibulum by the eighth week of gestation. The blood supply of the anterior and posterior pituitary gland comes from the bilateral superior and inferior hypophyseal arteries arising from the ICA [28]. It is not clear why pituitary hypoplasia would result from blocking of a unilateral blood supply and from abnormalities of the hypophyseal anatomy such as adenohypophysis hypoplasia and aplasia. However, the presence of ectopic neurophypophysis, which has been described in some patients, or other hypophyseal abnormalities, may also suggest a genetic etiology.

Pituitary gland development and function depend on the sequential temporal and spatial expression of multiple transcription factor genes such as *POU1F1* (*POU class 1 homeobox 1*; OMIM 173110), *PROP1* (*prophet of PIT1*; OMIM 601538), *HESX1* (*homeobox gene expressed in ES cells*; OMIM 601802), *LHX3* (*LIM homeobox gene 3*; OMIM 600577), *LHX4* (*LIM homeobox gene 4*; OMIM 602146), *SOX3* (*SRY-related HMG-box gene 3*; OMIM 313430), and *OTX2* (*orthodenticle homolog 2*; OMIM 600037) [4]. However, these defects may cause a deficiency in one or more pituitary hormones. Its clinical features vary in both severity and time of presentation, and onset may occur in the neonatal period or develop later in life [29–31]. In some patients, the hormonal deficiencies may present as a part of a syndrome with patients manifesting abnormalities in structures that share a common embryological origin with the pituitary gland [29–31].

In our review 6 patients, in addition to our, had a pituitary hypoplasia [3, 6, 8, 9, 11, 13]. This abnormality was isolated in two cases [6, 13], associated with ectopic neurohypophysis [9], absent pituitary stalk [8], or associated with a lack of posterior pituitary hyperintensity [3, 11]. However 5 patients had an absent adenohypophysis [5, 7, 10, 12] and 4 associated of these showed an ectopic neurohypophysis [5, 7, 10, 12].

Moreover, ectopic neurohypophysis may be accompanied by midline brain defects [32] supporting an embryological defect due to genetic factors [32, 33]. This has been reported in idiopathic GH deficiency or in subjects with *HESX1*, *LHX4*, and *SOX3* gene mutations [34]. For example, both the *SOX3* under- and over-expression may result in CPHD or IGHD in males, associated with infundibular hypoplasia, ectopic/undescended posterior pituitary, and abnormalities of the corpus callosum [35]. In these patients, congenital abnormalities of the ICA or other vessels have not been reported [35]. However, the associated midline anomalies (coloboma, single central maxillary tooth, transsphenoidal encephalocele, and Chiari I malformation) reported in some cases [5, 7, 9, 15] may support this hypothesis.

Of the patients in our review, one case had unilateral agenesis of the internal carotid artery associated with CPHD without *HESX1*, *LHX4,* or *OTX2* mutations [8]. Moreover, Savasta *et al.* [3] recently reported a CNV (copy number variant) i.e. a paternally inherited 200-kb deletion in 17q24.2. Thus, we performed array-CGH in our patient, but discovered no genomic imbalances. However, we indicate the need for further molecular analysis by NGS techniques to discover possible molecular abnormalities associated with this syndrome.

Given the rarity of the two conditions, the association is unlikely to be causal, although this cannot be excluded [36]. So, we hypothesize that an unknown gene mutation is the most likely explanation for the anatomical defects seen in these patients, suggesting a role for genetic factor(s) as a common cause of these two birth defects.

## Conclusions

Here were report the first case of a confirmed isolated GH deficiency in a patient with ICA agenesis. Our experience suggests the opportunity to carefully evaluate possible vascular abnormalities in case of IGHD or CPHD. The presence of an isolated pituitary deficit, and the rare association between the two conditions, suggest a genetic rather than a hemodynamic mechanism. Further studies, especially with NGS-based molecular analysis, could clarify this issue and the underlying mechanisms to better understand the etiopathogenetic characteristics of this disorder.

## Consent

Parents of the patient provided written informed consent for publication of this Case Report and any accompanying images. A copy of the written consent is available for review by the Editor of this Journal.

## Additional file

Additional file 1:
**CARE guidelines checklist of our Case Report.** (DOCX 14 kb)

## Abbreviations

ACTH: Adrenocorticotrophic hormone
CH: Congenital hypopituitarism
CPHD: Combined pituitary hormone deficiencies
GH: Growth hormone
GHD: Growth hormone deficiency
GHRH: Growth-hormone-releasing hormone
ICA: Internal carotid artery
IGHD: Isolated growth hormone deficiency
MRI: Magnetic resonance image
TSH: Thyroid-stimulating hormone
