# Supplementary material for: Agenesis of internal carotid artery associated with isolated growth hormone deficiency: a case report and literature review
Source: BMC Endocr Disord. 2015 Oct 19;15:58. doi: 10.1186/s12902-015-0037-y (PMC4617709; doi:10.1186/s12902-015-0037-y)
Supplement: Additional file 1: — CARE guidelines checklist of our Case Report. (DOCX 14 kb) [file 12902_2015_37_MOESM1_ESM.docx]

**CARE guidelines checklist of our Case Report**

|  | Ask | Response |
| --- | --- | --- |
| Title | The words “case report” (or “case study”) should appear in the title along with phenomenon of greatest interest (eg, symptom, diagnosis, test, intervention) | YES |
| Keywords | The key elements of this case in 2-5 words | YES |
| Abstract | 1. Introduction—What does this case add? | YES |
|  | 1. Case Presentation: The main symptoms of the patient | YES |
|  | The main clinical findings | YES |
|  | The main diagnoses and interventions | YES |
|  | The main outcomes | YES |
|  | 1. Conclusion—What were the main “take-away” lessons from this case? | YES |
| Introduction | Brief background summary of this case referencing the relevant medical literature | YES |
| Patient Information | 1. Demographic information (eg, age, gender, ethnicity, occupation) | YES |
|  | 1. Main symptoms of the patient (his or her chief complaints) | YES |
|  | 1. Medical, family, and psychosocial history—including diet, lifestyle, and genetic information whenever possible, and details about relevant comorbidities including past interventions and their outcomes | YES |
| Clinical Findings | Describe the relevant physical examination (PE) findings | YES |
| Timeline | Depict important dates and times in this case (table or figure) | YES |
| Diagnostic Assessment | 1. Diagnostic methods (eg, PE, laboratory testing, imaging, questionnaires) | YES |
| Diagnostic challenges | 1. (eg, financial, language/cultural) | YES |
|  | 1. Diagnostic reasoning including other diagnoses considered | YES |
|  | 1. Prognostic characteristics. (eg, staging) where applicable | YES |
| Therapeutic Intervention | 1. Types of intervention (eg, pharmacologic, surgical, preventive, self-care) | YES |
|  | 1. Administration of intervention (eg, dosage, strength, duration) | YES |
|  | 1. Changes in intervention (with rationale) | YES |
| Follow-up and Outcomes | Sumarize the clinical course of all follow-up visits | YES |
| Discussion | 1. The strengths and limitations of the management of this case | YES |
|  | 1. The relevant medical literature | YES |
|  | 1. The rationale for conclusions (including assessments of cause and effect) | YES |
|  | 1. The main “take-away” lessons of this case report | YES |
| Patient Perspective | The patient should share his or her perspective or experience whenever possible | YES |
| Informed Consent | Did the patient give informed consent? Please provide if requested | YES |
